# Effects of Patient-Selected Music Listening on the Pain and Anxiety of Patients Undergoing Hemodialysis: A Randomized Controlled Trial

**DOI:** 10.3390/healthcare9111437

**Published:** 2021-10-25

**Authors:** SukKyong Kim, HyeonCheol Jeong

**Affiliations:** 1 Department of Nursing, Sahmyook Medical Center, Seoul 01795, Korea; suk_kyung@naver.com; 2 College of Nursing, Sahmyook University, Seoul 01795, Korea

**Keywords:** hemodialysis, vascular access, music therapy, pain, anxiety

## Abstract

This study aimed to analyze the influence of patient-selected music listening on the pain and anxiety levels of hemodialysis patients after undergoing a vascular access operation. Methods: Sixty five patients were randomly assigned to the experimental group (*n* = 32) or the control group (*n* = 33). The experimental group was instructed to listen to their favorite music using headphones during their operations at the center. The control group underwent the operations without listening to any music. The pain measurement during vascular access operation was measured by subjective pain and objective pain behavior; anxiety was measured by subjective anxiety and anxiety states. Results: The experimental group reported significantly lower subjective pain levels than the control group (t = 9.36, *p* = 0.003). Regarding objective pain behaviors, the experimental group had a significantly lower score than the control group (t = 4.59, *p* = 0.036). The experimental group had significantly lowered subjective anxiety compared to the control group (F = 10.10, *p* = 0.002). Regarding anxiety states, the experimental group had significantly lower scores than the control group (F = 23.34, *p* < 0.001). Conclusion: The results suggest that patient-selected music listening reduced hemodialysis patients’ pain and anxiety levels during vascular access operations. Therefore, music medicine can be included as a new clinical intervention.

## 1. Introduction

According to the analyses, there are approximately 3.2 million patients with end-stage renal disease worldwide. Of these, 78% have received either hemodialysis or peritoneal dialysis and 22% have undergone kidney transplants [1]. With an increase in the aging population, there is also an increase in patients suffering from diabetes and high blood pressure, which are the main causes of terminal-stage kidney disease. In 2013, the number of hemodialysis patients was 69,837, an increase of 22.7% from the 2009 figure; the medical costs for treatment also increased by 32.2% within the same period. In 2013, there were 19,527 hemodialyzers, which indicated an increase of 42.5% from 2009 [2].

Reportedly, half the patients receiving dialysis treatment lived in North America, Europe, and Japan; terminal-stage kidney disease patients were most prevalent in Taiwan. Japan had 2525 patients per one million persons, and the United States (US) had 2138 [3]. Today, hemodialysis as a form of renal replacement therapy is a universal technique among chronic kidney disease patients that extends their overall life expectancy [4]. Hemodialysis is a life-saving procedure that requires the efficient removal and return of blood to the patient’s body [5].

Ever since the radiocephalic arteriovenous fistula (RC-AVF), as developed by Brescia, Cimino, and Appell, was released, vascular access operation techniques and vascular prostheses have improved through ceaseless research efforts. Vascular access creation has become a common surgical operation in the US, with more than 500,000 procedures performed during the past decade [6]. In addition, ongoing research aims to help hemodialysis patients improve their overall quality of life and reduce the complications related to arteriovenous fistula [7]. To manage the vascular access essential for hemodialysis patients, both medical staff and patients need to work harmoniously. However, vascular access operation can cause both pain and anxiety, and often causes patients to experience stress alongside the physical symptoms [8]. 

Music can affect the brain’s lymbic and autonomic nervous systems, causing emotional and physiological changes. Consequently, music can be used in the treatment of patients suffering from mental, emotional and physical pain [9]. How the music is used can be described as “music therapy” when it is used in the context of a therapeutic relationship with a certified music therapist, and can be described as “music medicine” when the medical effects are created by the music acting on the physical and psychological states of the patient, unmediated by a therapist. Therefore, both music therapy and music medicine use music to achieve therapeutic goals and to restore, maintain, and promote mental and physical health, and are economical and effective interventions without side effects [10]. In the study described in this report, patients selected their preferred music to use during the experimental procedure and so can be described as music medicine. Consequently, the term “music medicine” will be used to refer to the treatment involving patient-selected music listening.

When patients listen to their preferred music for pain reduction helps to reduce the perception of pain [9]. Regarding the selection of music, music that the subject does not enjoy may act as noise and induce stress, which increases blood pressure, pulse, and anxiety [11]. For this reason, music interventions with patients’ preferred music are diversely used as a non-invasive therapy that can be easily performed in clinical settings for patients with simple surgery [12,13]. For example, when hemodialysis was applied to patients, listening to their favorite music was found to be significantly effective in reducing both their fatigue and anxiety levels [14]. In one study, female college students who listened to their favorite music showed a significant decrease in anxiety compared to those who did not listen to music [11]. As such, the pleasure that participants felt when listening to their favorite music had a positive effect on lowering their stress levels [15,16]. However, discomfort is distinguished from neutral or pleasurable emotions by the activation of the bilateral occipital-temporal cortex, cerebellum, left hippocampal gyrus, hippocampus, and amygdala [17]. Chronic psychological or physical stress is often associated with chronic pain, but this relationship is poorly understood. In this relationship, the output patterns of the body–self neuromatrix activate perceptual, homeostatic, and behavioral programs after injury, pathology, or chronic stress [18]. 

Listening to music was shown to alleviate the pain, anxiety, and depression of gynecologic cancer patients who had received chemotherapy [19]. When music therapy was applied to gastroscopy and cystoscopy patients, it was significantly effective in reducing their anxiety and pain levels [20,21].

Previous studies suggested that when participants listened to music they enjoyed, music medicine proved to be an effective tool in reducing their physical and mental stress, as well as increasing their safety and pain thresholds [22]. However, little research is available on the effect of music medicine as an intervention for dialysis patients’ vascular access operation-related complications. Therefore, this study attempted to uncover the effects of music medicine as a non-pharmacological treatment to develop a clinical intervention to reduce both the anxiety and pain experiences of hemodialysis patients with vascular access dysfunctions after they had undergone vascular access operations.

## 2. Materials and Methods

### 2.1. Participants

From June to October 2018, 142 patients undergoing vascular access surgery at S General Hospital in Seoul were given handouts and explanations regarding the recruitment of study subjects, and then those who agreed to participate in this study were selected. To calculate the sample size, we used the analysis method of ANCOVA, with effect size of 0.4, significance level of 0.05, and power of 0.8. The result was that at least 52 participants were needed. We selected a total of 65 participants, as we anticipated a 20% drop out rate. There were no drop outs, therefore 65 participants were analyzed. After giving temporary numbers to 65 participants, the randomization site program (http://www.randomization.com accessed on 15 June 2018) was used to randomize the experimental group (32 people) and the control group (33 people).

Participation criteria:

- Subjects who will undergo vascular access surgery, among patients undergoing hemodialysis;
- Subjects who can express their opinions clearly and communicate;
- Subjects with no hearing problems;
- Subjects who agreed to participate in the experiment;
- Subjects who did not receive psychotropic drugs or sedatives during the procedure.

### 2.2. Procedure

**Figure 1:**
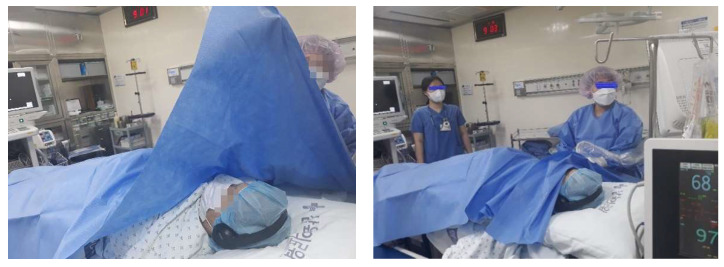
Vascular access surgery with music therapy.

The experimental group was instructed to listen to their favorite music using a Bluetooth headset during vascular access surgery. In this study, as a preparatory process for music therapy, the types of music that each subject liked were investigated in advance. Based on this, after paying for the official sound source on the Internet ‘Melon’ music site, it was downloaded to a mobile phone or MP3 player. The number of preferred songs required for the 30 min intervention was about 10 to 12 songs. During the vascular access surgery procedure, the experimental group used Bluetooth headphones to listen to their preferred music so as not to interfere with the procedure (Figure 1). The volume level was maintained at a level that allowed communication to be carried out while listening to music. The control group performed vascular access surgery in a quiet environment without music intervention. The detailed music medicine procedure is shown in (Table 1).

### 2.3. Measurement

#### 2.3.1. Pain Measurement Tool

- Subjective pain

Pain is defined as an unpleasant sensory and emotional experience related to either actual or potential tissue damage. Using the Visual Analog Scale (VAS), this study measured a value that signified the degree of pain along a straight line of 10 cm. The higher the score, the more severe the pain experienced.

- Checklist of objective pain behaviors

To measure patients’ pain levels, an objective pain behavior checklist, as developed by Park [23], was utilized. This checklist focuses on facial expressions (10 items, range of 0–3 points), voice changes (8 items, range of 0–4 points), and the degree of perspiration (11 items, range of 0–2 points). The sum of the checklists ranged from 0 to 9, with higher scores indicating more severe pain. 

#### 2.3.2. Anxiety Measurement Tool

- Subjective anxiety

Subjective anxiety was measured using the VAS scale and rated on a scale from 0 to 10, with 0 indicating no anxiety and 10 indicating severe anxiety.

- Anxiety states

Mood Status Profile Tool (POMS), modified by Lee [24] and based on the scale developed by McNair et al. [25], was used to measure anxiety. This anxiety measure consisted of nine items (tension, nervousness, shakiness, feeling on edge, panic, uneasiness, restlessness, anxiety, and feeling relaxed), which were rated on a four-point Likert scale. The higher the overall score, the greater the anxiety state experienced. Regarding the reliability of this scale in this study, Cronbach’s α = 0.92.

### 2.4. Analysis

The collected data were analyzed using the SPSS Ver.25 program. The study results were analyzed using an independent t-test. In addition, to increase the accuracy of the analysis results, ANCOVA, in which the previous score was controlled as a covariate, was used. The reliability of the tool was verified by Cronbach’s α coefficient.

## 3. Results

### 3.1. General Characteristics and the Homogeneity Test

A homogeneity test was conducted before participants underwent their operations to ensure that the control and experimental groups were homogeneous in terms of age, sex, number of vascular access operation, vascular types, periods of vascular use, music preferences, subjective anxiety levels, and anxiety states (Table 2).

### 3.2. The Effect of Music Medicine on Subjective Pain

Regarding subjective pain, the experimental group (3.38 ± 2.61) showed a significantly lower score than the control group (5.55 ± 3.55) (t = 9.36, *p* = 0.003). Therefore, music medicine reduced the patient’s subjective pain level during the vascular access surgery procedure (Table 3).

### 3.3. The Effect of Music Medicine on Objective Pain Behaviors

Regarding objective pain behaviors, the experimental group (2.97 ± 2.81) had a significantly lower score than the control group (4.67 ± 3.49 points) (t = 4.59, *p* = 0.036). Therefore, the application of music medicine to patients undergoing vascular access surgery decreased the objective pain behavior (Table 4).

### 3.4. The Effect of Music Medicine on Anxiety

The analysis of subjective anxiety indicated that the experimental and control groups had an average score of 2.59 ± 1.80 and 3.42 ± 3.37, respectively. This showed that the experimental group, which experienced the music therapy, had a significantly lowered subjective anxiety than the control group (F = 10.10, *p* = 0.002). 

**Figure 2:**
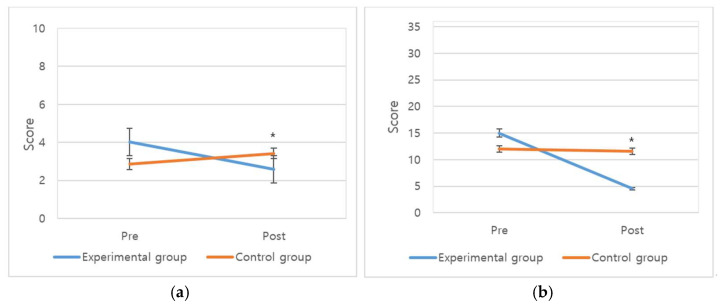
The effects of music medicine on anxiety: (**a**) Difference between before and after music medicine for subjective anxiety; (**b**) Difference between before and after for anxiety state (POMS) after music medicine. POMS: Profile of Mood States, * *p* < 0.05.

The analysis of objective anxiety states indicated that the scores of the experimental group (4.53 ± 4.50) were significantly lower than that of the control group (11.55 ± 12.17) (F = 23.34, *p* < 0.001) (Table 5 and Figure 2).

## 4. Discussion

This study analyzed the effects of music medicine on the pain experienced by hemodialysis patients who underwent vascular access operations. Our findings indicated that the experimental group, which experienced music therapy, demonstrated significantly lower subjective pain levels than the control group. 

This study was similar to the findings in Hosseini’s study [26] that music therapy was effective in reducing pain in mothers, and in Cho’s study [8] that hand massage was effective in reducing pain in hemodialysis patients. In Jeong’s study [27], when patients who had undergone total knee arthroplasties listened to their favorite music during manual joint movements, their pain was significantly reduced. These results indicate that pain levels can be managed not only by medications, but also through music therapy.

In the present study, the effect of music medicine on patients’ anxiety levels was analyzed. The results indicated that the experimental group had significantly lower subjective and objective anxiety compared to the control group. In previous studies, music medicine was found to be effective at reducing the anxiety of patients who had undergone a surgical operation under localized anesthesia [20,28]. In the study conducted by Park [29], when coronary angiography patients listened to their favorite music, their overall anxiety states were reduced. 

According to a study by Son et al. [30], the experimental group of patients, who listened to music for 30 min when a burn patient had to put a dressing on their wound, had lower anxiety than the control group. According to a study by Kim [31], the experimental group in which music medicine was applied to hemodialysis patients was more effective in reducing anxiety than the control group. The results of these previous studies are similar to the results of this study and support that music medicine is effective in reducing anxiety.

On the other hand, according to Kwon’s study [32] in which music intervention was applied to surgical patients at a military hospital, there was no significant difference in anxiety levels, which was different from the results of this study. It is thought that a short period of music intervention (15 min) listened to in the operating waiting room was insufficient to reduce the anxiety of surgery. In addition, because the subjects were soldiers, the characteristics of their occupations were different from those of civilians, because they experienced life in a more anxious state. Additionally, it is thought that the music in this study was ineffective because it was a type of music (Brandenburg Concerto) that they did not normally listen to.

Overall, music medicine was found to be effective in reducing pain and anxiety during vascular access in hemodialysis patients with vascular channel dysfunction. Pain is produced by the output of a widely distributed neural network in the brain. The neuromatrix, which is genetically determined and modified by sensory experience, is the primary mechanism that generates the neural pattern that produces pain [18].

Music medicine is an effective nursing intervention that is economical and has no side effects in restoring the patient’s mental and physical health when performing simple procedures in the hospital. Based on this, the continuously repeated research on music therapy, which is a positive and new intervention for reducing pain and anxiety in patients, is necessary. 

## 5. Conclusions

According to the findings of this study, music medicine positively influenced the subjective and objective pain behaviors, subjective anxiety levels, and anxiety states of hemodialysis patients with vascular access dysfunctions during their vascular access surgery procedures. The research results can inform new clinical interventions to reduce pain and anxiety in patients. In light of this, it is necessary to conduct continuous and repeated research studies on music medicine within clinical settings when hemodialysis patients undergo vascular access surgery. 

## Figures and Tables

**Table 1:** Music medicine process.

Music Medicine Process	Description
Pre-test	Received written agreement after an explanation of the research, before undergoing their operation. Surveyed their favorite music by genre (classical, religious, pop, and others), title, and artist.
Music selection	Purchased their favorite music sources from an online music pay site, and then saved them on an MP3 player. The music used for vascular access surgery consisted of 10-12 songs played for 30 min.
Music medicine (30 min from the start)	The participants put on the Bluetooth headphones at the beginning of their vascular access surgery, which did not impede the operation process. The experimental group listened to their favorite music for the duration of their operations (around 30 min), with the volume of the music remaining at a communicable level. The control group underwent their vascular access surgery in a quiet environment without receiving any music therapy.
Post-test	At 30 min following the vascular access operation, three nurses at the center recorded the patient’s objective pain indices (facial expressions, voice changes, and degrees of perspiration). Both the experimental and control groups partook in a questionnaire survey measuring their subjective pain levels, subjective anxiety, and overall anxiety states.

**Table 2:** General characteristics and the homogeneity (*N* = 65).

General Characteristics		Experimental Group (*n* = 32)	Control Group (*n* = 33)	X^2^/*t*	*p*
		*n* (%) or M ± SD	*n* (%) or M ± SD		
Age (year)	<60	9 (28.1)	10 (30.3)	0.04	0.982
	60–69	9 (28.1)	9 (27.3)		
	70≤	14 (43.8)	14 (42.4)		
Gender	Male	19 (59.4)	23 (69.7)	0.76	0.443
	Female	13 (40.6)	10 (30.3)		
Vascular access operation (rate)	None	15 (46.9)	20 (60.6)	2.43	0.296
	1	7 (21.9)	8 (24.2)		
	2≤	10 (31.3)	5 (15.2)		
Vascular types	Arteriovenous fistula	11 (34.4)	8 (24.2)	0.80	0.422
	Artificial blood vessel	21 (65.6)	25 (75.8)		
Periods of vascular use (year)	≤1	6 (18.8)	9 (27.3)	0.67	0.716
	1–2	11 (34.4)	10 (30.3)		
	2≤	15 (46.9)	14 (42.4)		
Music preferences	Very bad	0 (0)	1 (3)	4.28	0.118 *
	Neither	4 (12.5)	10 (30.3)		
	Very good	28 (87.5)	22 (66.7)		
Music type	Gospel	5 (15.6)	3 (9.1)	4.14	0.126 *
	K-pop	26 (81.3)	24 (72.7)		
	Country- Western	1 (3.1)	6 (18.2)		
Subjective anxiety levels (score)		4.03 ± 2.04	2.85 ± 2.71	1.98	0.052
Anxiety states (score)		14.97 ± 8.76	12.00 ± 9.83	1.28	0.204

* Fisher’s exact test.

**Table 3:** The effect of music medicine on subjective pain.

Experimental Group (*n* = 32)	Control Group (*n* = 33)	t	*p*
M ± SD	M ± SD		
3.38 ± 2.61	5.55 ± 3.55	9.36	0.003

**Table 4:** The effect of music medicine on objective pain behaviors.

Categories	Experimental Group (*n* = 32)	Control Group (*n* = 33)	t	*p*
	M ± SD	M ± SD		
Facial expression	0.97 ± 0.97	1.85 ± 1.58	6.41	0.014
Voice change	1.41 ± 1.47	1.97 ± 1.74	4.22	0.206
Perspiration level	0.50 ± 0.76	0.85 ± 0.834	2.94	0.091
Total	2.97 ± 2.81	4.67 ± 3.49	4.59	0.036

**Table 5:** The effect of music medicine on anxiety.

Categories		Pre Test M ± SD	Post Test M ± SD	Ajusted M ± SD	F *	*p*
Subjective anxiety	Experimental group	4.03 ± 2.04	2.59 ± 1.80	3.60 ± 1.30	10.10	0.002
	Control group	2.85 ± 2.71	3.42 ± 3.37	12.45 ± 1.28		
Anxiety states (POMS)	Experimental group	14.97 ± 8.76	4.53 ± 4.50	2.16 ± 0.37	23.34	<0.001
	Control group	12.00 ± 9.83	11.55 ± 12.17	3.85 ± 0.37		

*: ANCOVA results with covariate pre-test. POMS: Profile of Mood States.

## Data Availability

Publicly available datasets were analyzed in this study. This data can be found here: http://www.jerrydallal.com/random/randomize.htm accessed on 15 June 2018.
